# An Analog Interface Circuit for Capacitive Angle Encoder Based on a Capacitance Elimination Array and Synchronous Switch Demodulation Method

**DOI:** 10.3390/s19143116

**Published:** 2019-07-15

**Authors:** Bo Hou, Bin Zhou, Xiang Li, Zhenyi Gao, Qi Wei, Rong Zhang

**Affiliations:** Engineering Research Center for Navigation Technology, Department of Precision Instrument, Tsinghua University, Beijing 100084, China

**Keywords:** angle encoder, capacitance elimination array, switch synchronous demodulation method, application-specific integrated circuit

## Abstract

This paper presents an analog interface application-specific integrated circuit (ASIC) for a capacitive angle encoder, which is widely used in control machine systems. The encoder consists of two parts: a sensitive structure and analog readout circuit. To realize miniaturization, low power consumption, and easy integration, an analog interface circuit including a DC capacitance elimination array and switch synchronous demodulation module was designed. The DC capacitance elimination array allows the measurement circuit to achieve a very high capacitance to voltage conversion ratio at a low supply voltage. Further, the switch synchronous demodulation module effectively removes the carrier signal and greatly reduces the sampling rate requirement of the analog-to-digital converter (ADC). The ASIC was designed and fabricated with standard 0.18 µm CMOS processing technology and integrated with the sensitive structure. An experiment was conducted to test and characterize the performance of the proposed analog interface circuit. The encoder measurement results showed a resolution of 0.01°, power consumption of 20 mW, and accuracy over the full absolute range of 0.1°, which indicates the great potential of the encoder for application in control machine systems.

## 1. Introduction

Measuring the angular position is important in the automotive industry [1,2,3,4,5]. Angle sensors are widely used in unmanned aerial vehicles (UAVs), small robots, and high-precision gimbal systems. These applications require angle sensors to have a small size, light weight, low power consumption, and low cost [6,7,8,9,10].

Angular position sensors commonly available in the market include gratings and inductive synchronizers [11,12]. These sensors have high precision but have the disadvantages of large volume, complicated processing circuit, high cost, and high-power consumption. They are difficult to apply in fields such as drone robots and UAVs. In view of these shortcomings, many angular displacement sensors based on the principles of capacitance and inductance of printed circuit board (PCB) technology have been presented [6,13,14,15,16,17]. These sensors have a simple structure, low manufacturing cost, small size, and high precision with a greatly reduced volume compared to gratings and magnetic grids. Capacitive encoders have been gaining interest due to their simple design, potential for further miniaturization, and insensitivity to magnetic field variations.

Karali et al. [14] developed new economical capacitive rotary encoder based on analog synchronous demodulation. The carrier signal is removed by multiply demodulation in the analog system, which greatly reduces the sampling rate requirement for the analog-to-digital converter (ADC). The overall system is cheaper than digital counterparts. However, the demodulation circuit is sensitive to temperature and requires the two analog blocks to be highly symmetric. During demodulation, synchronous and quadrature demodulation techniques are widely used, especially for applications requiring high levels of performance in harsh environments [13]. The capacitive encoder produces a phase/frequency modulated signal that cannot be demodulated by the traditional amplitude demodulation techniques of resolvers. With the increase in embedded technologies, digital-based demodulation techniques are becoming common in the literature. The central digital computational block can be a fast microcontroller or a field programmable gate array (FPGA)-based embedded system. Hou et al. [18] presented a novel single-excitation capacitive angular position sensor (CAPS) that outputs two amplitude modulated signals, which can be demodulated by traditional amplitude demodulation techniques such as resolvers. However, the traditional resolver demodulation chip has a high-power consumption and poor expansion as nonlinear error information of the sensor is difficult to observe and obtain. Further, because of the presence of the DC capacitance $C_{0}$, the swing amplitude of the output signal is limited. This results in a high-power supply voltage to achieve a high capacitance resolution.

Many demodulation schemes are available for angle sensors [19,20,21,22], but there is no integrated circuit that has been specifically designed for it. The common capacitive angular displacement sensor usually needs a high supply voltage which leads to large power consumption, and the demodulation circuit is complex and difficult to integrate. It is urgent to design an analog-interfacing circuit to effectively solve the problem of high power consumption, large size, and difficult integration of the encoder demodulation circuit.

This paper proposes a simple but effective input interfacing scheme that uses a capacitance elimination array and the switch synchronous demodulation method. To achieve miniaturization, low power consumption and easy integration, an analog interface circuit that includes a DC capacitance elimination array and switch synchronous demodulation module was designed. The DC capacitance elimination array allows the measurement circuit to achieve very high capacitance to voltage ratios at a low supply voltage. The switch synchronous demodulation module effectively removes the carrier signal and greatly reduces the sampling rate required by the ADC.

## 2. Capacitive Angular Encoder

The encoder is composed of two main parts: a stator and rotor. Capacitive rotary encoders are based on a rotating disk on a fixed plate or between two fixed plates stator and rotor. As an encoder rotates, the capacitance between stator and rotor change, and the angle of the shaft can be extracted. The rotor has been printed a petal-form sensitive electrode and a coupling electrode. The stator has been printed one excitation electrode and four sets of collection electrodes. The petal-form shape sensitive electrode on rotor is connected with coupling electrode. The sensor is excited by a square wave voltage. The carrier excitation voltage is applied to the stator excitation electrodes:

(1) $$U_{E}=A_{E}\cdot squ(\omega t)$$

where ω is the frequency of the excitation voltage, $A_{E}$ is the amplitude and *squ*(·) means square wave function.

Via capacitive coupling between the rotor and stator, a four-way modulation of the angular information capacitance signal can be obtained on the collector electrode of the stator:

(2) $$\left\{ \begin{array}{l} U_{S+}=k\cdot (C_{0}+\Delta C\sin(\phi ))\cdot squ(\omega t)\\ U_{S-}=k\cdot (C_{0}-\Delta C\sin(\phi ))\cdot squ(\omega t)\\ U_{C+}=k\cdot (C_{0}+\Delta C\cos(\phi ))\cdot squ(\omega t)\\ U_{C-}=k\cdot (C_{0}-\Delta C\cos(\phi ))\cdot squ(\omega t) \end{array}\right.$$

where $C_{0}$ is the DC capacitance, ϕ is the modulated angle information, and ΔC is the magnitude of the useful angular signal information. $U_{S+}$, $U_{S-}$, $U_{C+}$, and $U_{C-}$ are the voltage signals after being converted by the capacitance–voltage (C–V) model of the four detection capacitors. k is the ratio of C–V model. The existence of the DC capacitance limits the swing amplitude of the output signal. The differential sensitivity principle is more effectively reduce common mode interference.

Two differential amplifier modules are used to eliminate the DC component output. Finally, two orthogonal amplitude modulated signals $U_{S}$ and $U_{C}$ can be obtained.

(3) $$\left\{ \begin{array}{l} U_{S}=A\cdot \Delta C\sin(\phi )\cdot \sin(\omega t)\\ U_{C}=A\cdot \Delta C\cos(\phi )\cdot \sin(\omega t) \end{array}\right.$$

A capacitive encoder structure was designed and fabricated with PCB technology to test the proposed analog interfacing circuit. Based on the design diameters, the DC capacitance $C_{0}$ was 1 pF, and the sensitive capacitance ΔC was approximately 1 pF.

## 3. Signal Processing of the ASIC

### 3.1. Processing Circuit Introduction

**Figure 1:**
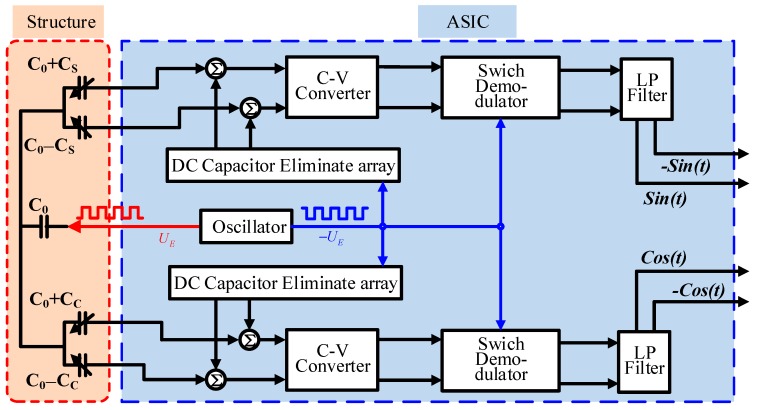
Block diagram of the proposed architecture.

The integrated analog-interfacing circuit includes the DC capacitance elimination array, C–V conversion module, switch synchronous demodulation module, and filter. Figure 1 shows a block diagram of the proposed architecture. During operation, an external oscillator generates two differential excitation signals: $U_{E}$ and $-U_{E}$. The positive signal $U_{E}$ acts on the capacitive encoder, and the negative $-U_{E}$ accesses the DC capacitance cancellation array. When the sensor is working, the DC capacitance $C_{0}$ of the four-way measurement capacitance can be eliminated by combining the capacitance elimination array and negative signal $-U_{E}$. The differential output charge amplifier converts the change in capacitance to an equivalent voltage. Then, the switch demodulation module is used to eliminate the carrier signal. The related voltage signal is further filtered and amplified by the low pass filter module. Then, the voltage is quantized to obtain a digital signal. The presented circuit can effectively detect the capacitance change of capacitive angle encoder and is applicable in a wide range of size and capacitance value of encoder.

### 3.2. On-Chip DC Capacitance Elimination Array

The on-chip capacitor array incorporated on the ASIC is purposefully to eliminate the DC capacitance $C_{0}$ and compensate for the inconsistency of the two sensitive capacitors. Equation (1) shows that there is a DC $C_{0}$ for the four-measurement capacitance. Therefore, if $C_{0}$ cannot be eliminated, the supply voltage (*V_DD_*) cannot fully applied to the sensing capacitance ΔC. $C_{0}$ severely restricts the output signal swing. To compensate this offset, two identical sets of on-chip parallel DC capacitors as elimination array are included within the ASIC. Each set is connected to either $C_{S+}$ or $C_{S-}$, whichever value is controlled by external pins.

An external oscillator generates two differential excitation signals $U_{E}$ and $\bar{U_{E}}$. $U_{E}$ acts on the capacitive angular encoder as a carrier excitation signal, and $\bar{U_{E}}$ accesses the DC capacitance elimination array. The two differential excitation signals can be expressed as

(4) $$U_{E}=A_{E}\cdot squ(\omega t)$$

(5) $$\bar{U_{E}}=-A_{E}\cdot squ(\omega t)$$

**Figure 2:**
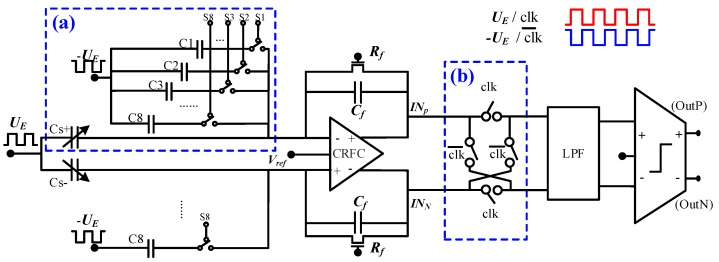
DC cancellation capacitor and switching demodulation: (**a**) capacitance elimination array and (**b**) synchronous switch demodulation.

The DC capacitance cancellation array is used to cancel the DC capacitance of the four sensing capacitors. Normally, the capacitance error of the sensor is completed after processing, so the design of this module enables the DC error of the sensor to be corrected. In the design, the eight control switches S1, S2, S3, …, S8 are used to control the value of elimination capacitors. In the capacitance elimination array, the capacitance value of single bit is *C*, and switch S*n* is corresponding to a capacitor of 2*^n^C* (*n* = 1, 2, …, 8), so the value of each elimination capacitor ranges from 0 to $2^{8}\cdot C$. The main connection relationship is shown in Figure 2. In the design, *C* was set to 49 fF.

If the value of elimination capacitors is $C_{ELI}$, the charge on the C–V conversion can be expressed as

(6) $$\begin{array}{ll} Q & =CU=C_{S+}\cdot k\cdot A_{E}\cdot squ(t)+C_{ELI}\cdot (-A_{E}\cdot squ(t))\\ & =[(C_{0}+\Delta C\sin(\phi ))\cdot k-C_{ELI}]\cdot A_{E}\cdot squ(t) \end{array}$$

where k is the conversion ratio and is determined by sensitive structure. This operation tries to adjust the capacitance $C_{ELI}$ to ensure $C_{0}\cdot k=C_{ELI}$.

Then, Equation (6) can be simplified as

(7) $$Q=(\Delta C\sin(\phi ))\cdot k\cdot A_{E}\cdot squ(t)$$

According to the output function of the charge amplifier,

(8) UO=−jωAQ(1+A)(1Rf+jωCf)

Thus, the output voltage is only determined by the input charge and the parameters of the feedback circuit ($R_{f}$, $C_{f}$). Further, when $R_{f}$ is large enough to satisfy $1/R_{f}<<\omega \;C_{f}$ ($R_{f}=10^{8}\;\Omega$ in this paper), $1/R_{f}$ can be omitted. The output can be expressed as

(9) $$U_{O}=\frac{-j\omega AQ}{(1+A)j\omega C_{f}}=-\frac{AQ}{(1+A)C_{f}}\approx -\frac{Q}{C_{f}}$$

The output voltage is only related to the charge Q and feedback capacitance $C_{f}$:

(10) $$IN_{P}=\frac{\left(\Delta C\sin(\phi ))\cdot k\cdot A_{E}\cdot squ(t\right)}{C_{f}}$$

(11) $$IN_{N}=-\frac{\left(\Delta C\sin(\phi ))\cdot k\cdot A_{E}\cdot squ(t\right)}{C_{f}}$$

**Figure 3:**
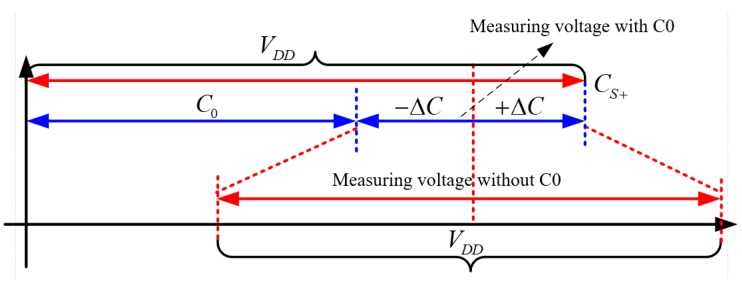
Effect of the offset capacitance on the scaling factor.

If *C*_0_ exists in the circuit, then after it enters the charge amplifier, because of the limited power supply voltage *V*, using the cancellation capacitor can ensure that the scale factor of the capacitor-voltage ratio is maximized at low voltage, as Figure 3 shows. This can effectively suppress the DC error, and precision measurement with a low supply voltage can be easily achieved.

**Figure 4:**
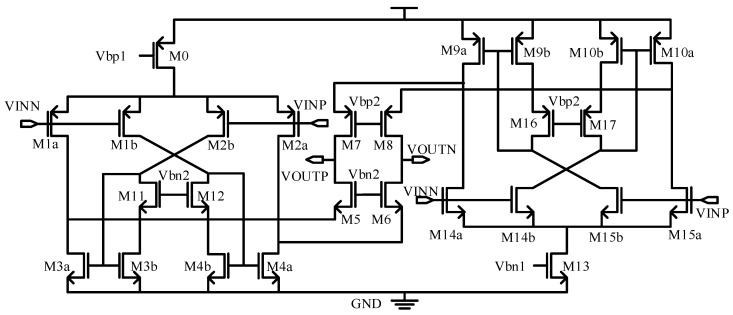
The structure of CRFC operational transconductance amplifier (OTA).

The amplifier in this C–V conversion circuit adopts the complementary recycling folded cascode (CRFC) architecture [23] due to its larger gain-bandwidth-product (GBW) and slew rate compared with conventional folded cascode op-amp as shown in Figure 4. It has NMOS and PMOS transistors as complementary input differential pairs, and in both branches, the recycling folded cascode (RFC) technique [24] is introduced to achieve more biasing current efficiency. The CRFC amplifier improves its GBW by a factor of 1/3 compared with the RFC under the same biasing current and capacitive load.

### 3.3. Synchronous Switch Demodulation and Low-Pass Filter

**Figure 5:**
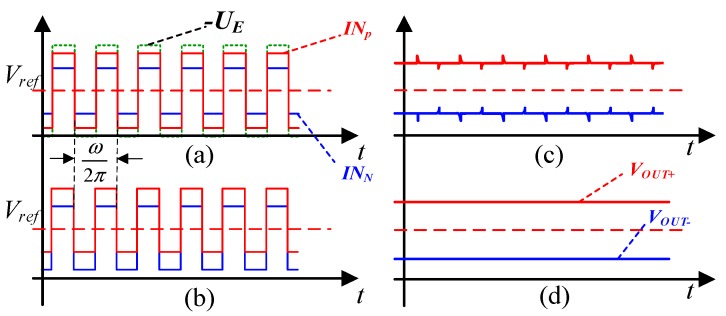
Diagram of the synchronous switch demodulation method: (**a**) two signals after the C–V conversion module and switching signal; (**b**) output signal of *IN_P_* and *IN_N_* after the switch demodulated; (**c**) Signals after differential; and (**d**) the differential signal is output after filtering.

In the proposed configuration, the signals *IN_P_* and *IN_N_* is demodulated by the switch demodulation module. The demodulation process is shown in Figure 5. The demodulated output signals are then buffered and fed to the low-pass filter. In this design, a filter which owns the cutoff frequency on 10 kHz has been applied to reduce noise of switches. The low-pass filter provides an analog output corresponding to the input angle information being fed to the ADC.

The voltage signal after synchronous switch demodulation is given by

(12) $$U_{InP}=U_{P}-U_{N}=\frac{2\cdot (\Delta C\sin(\phi ))\cdot k\cdot A_{E}}{C_{f}}+Noise_{SW}$$

where $Noise_{SW}$ is the noise due to the synchronization error of the switch demodulation, as shown in Figure 5c. It can be decreased after the low-pass filter.

In order to achieve a supply voltage of 5 V, a reference voltage *V_ref_* of 2.5 V is applied to the analog circuit. The output signal after the bandpass filter (BPF) can be expressed as

(13) $$V_{OUT+}=V_{ref}+\frac{2\Delta C}{C_{f}}V_{P-P}$$

where $V_{P-P}$ is the peak-to-peak amplitude of the excitation signal. Similarly, because of the fully differential nature of the operational transconductance amplifier (OTA), $V_{OUT-}$ can be written as

(14) $$V_{OUT-}=V_{ref}-\frac{2\Delta C}{C_{f}}V_{P-P}$$

Thus, the final differential output value can be obtained:

(15) $$\Delta V_{OUT}=\frac{4\Delta C}{C_{f}}V_{P-P}$$

The sensitivity of the proposed configuration is double that of conventional architectures. In general, *V_P−P_* is equal to the supply voltage (*Vcc*) of the system. The gain of the charge amplifier can be programmed by selecting the proper feedback capacitance *C_f_*. In this design, three control pins provide eight programmable gains.

## 4. Measurement Results

### 4.1. Characteristics Test of the ASIC

**Figure 6:**
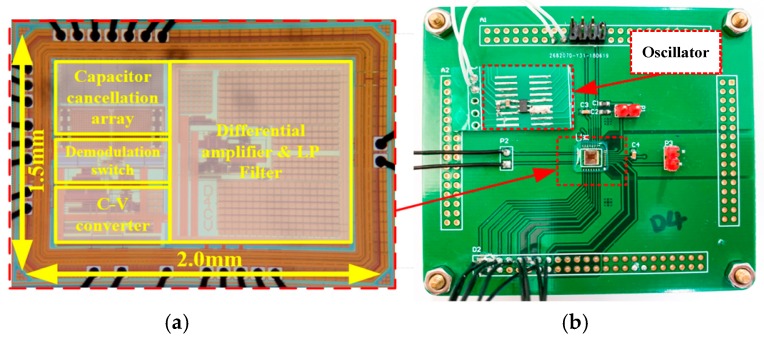
ASIC circuit test chart: (**a**) microphotograph of the circuit chip and (**b**) test circuit diagram.

The proposed analog interface circuit was designed and fabricated with SMIC 0.18 µm CMOS processing technology. A microphotograph of the chip is shown in Figure 6a. The scale factor, offset capacitance, zero offset stability, and noise level of the ASIC chip have been tested. The test circuit diagram and chip layout are shown in Figure 6b. An oscillator chip was used to generate the carrier signal to actuate the encoder.

#### 4.1.1. Scale Factor

**Figure 7:**
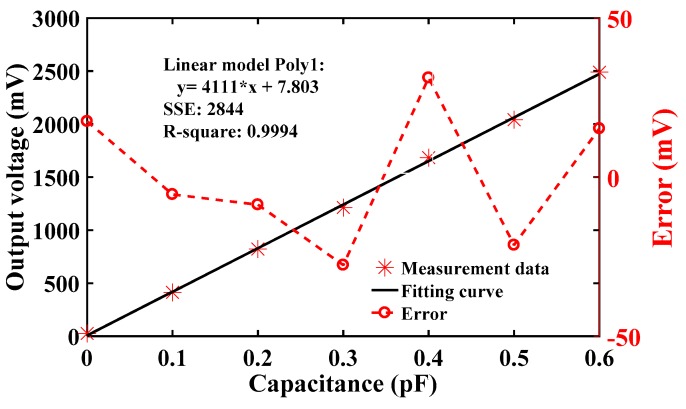
Relationship between the output voltage and access capacitance.

In the scale factor test experiment, the measured capacitance was changed and increased in increments of 0.1 pF. The capacitors 0.1 pF, 0.2 pF, 0.3 pF, et al., was picked out via capacitance measurement instrument. Then, the capacitors are replaced to the circuit board in turn. The connected capacitor should be increased in increments of 0.1 pF without consider the parasitic capacitance. The output voltage was measured, and the data were imported into MATLAB to graph the results, as shown in Figure 7. The linearity was excellent, and the slope was 4.111 V/pF, which is basically consistent with the design value. The residual is shown as the red line. This was mainly because the value of the connected capacitance could not be accurately determined, so the linearity of the measured value had a residual of about 30 mV. This test proved that the design functioned correctly.

#### 4.1.2. Noise Test

**Figure 8:**
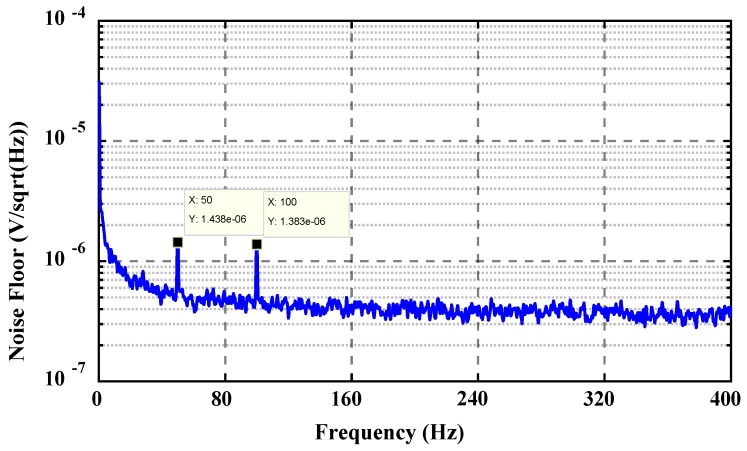
Noise spectrum of the ASIC voltage output.

Next, the noise of the circuit was tested. The output noise of the interface circuit was tested with 35670A produced by Agilent Technologies Inc. The noise spectrum of the output voltage is shown in Figure 8. The floor for the output voltage noise of the signal was 400 nV. The points marked in the figure indicate a power frequency interference of 50 Hz. This would have a strong influence and needs to be reduced by careful shielding during operation. In addition, the noise also exhibits a strong 1/*f* characteristic, mainly due to the low-pass filter after switching demodulation.

The capacitance resolution *R_C_* was calculated according to the noise floor and conversion coefficient:

(16) $$R_{C}=N_{f}/S_{C}$$

Here, the calculation of the capacitance resolution was affected by two main factors. The previous test result showed that $N_{f}=400\;\mathrm{nV}$ and $S_{C}=4.111\;V/\mathrm{pF}$. According to Equation (16), the capacitance resolution of the circuit was 10^−19^ F. The capacitance of the micro-sensitive structure described above varied from 0.3 to 0.5 pF. Thus, the circuit could satisfy the demodulation of the angular position information of the sensitive structure.

### 4.2. Characteristics Test of Encoder

**Figure 9:**
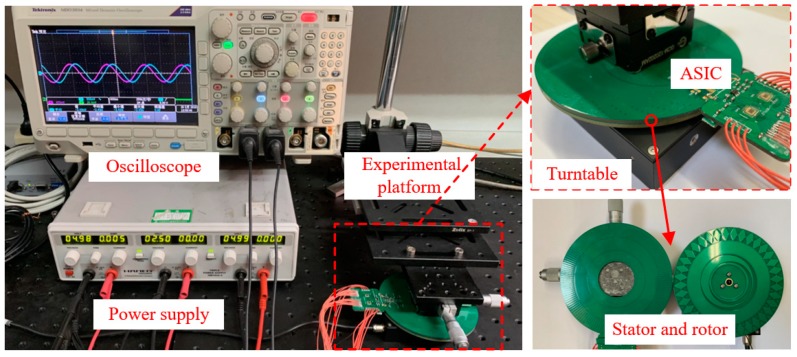
Experimental setup to test the encoder. The setup includes a high precision turntable, a set of fixing device, a rotor and a stator. The fixing device is used to fix the processing circuit to the backside of the stator.

The analog interface circuit was connected with the sensitive structure for further testing. Figure 9 shows the experimental setup for testing the characteristics of the encoder. The setup consisted of a high-precision turntable and sensor mounting system. For the sensor installation, the gap was mainly controlled by the feeler gauge to ensure clearance and concentricity. The gap between the stator and rotor was 0.3–0.5 mm. The concentricity was determined by marks at the edges of the rotor and stator to achieve alignment. As shown in the figure, the device mainly consisted of two parts: the fixed system of the rotor and stator, which needed five degrees of freedom to achieve the above adjustment of the eccentricity and tilt; and a miniature precision turntable purchased from Physik Instrumente Corporation. The diameter of the turntable was 23 mm, and the position accuracy was 0.005°. The stator and the rotor plates, both of which are fabricated by advanced PCB technology with the diameter of 54 mm.

#### 4.2.1. Angle Stability Test

**Figure 10:**
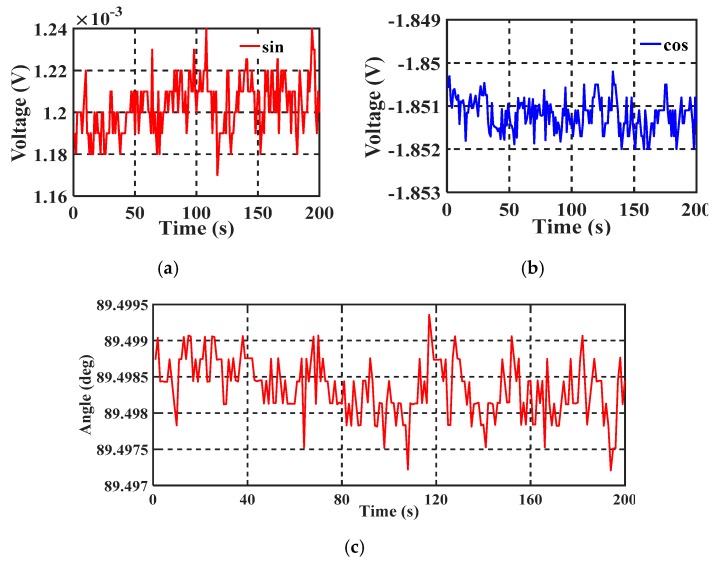
Stability measurement results: (**a**) and (**b**) show the stable voltage output and (**c**) shows stable angle output.

The long-term stability of the encoder was tested. Keithley 2010 was used to collect the output voltage data, which were then saved and processed with a computer. Figure 10a,b show the long-term voltage stability of two orthogonal signals. The voltage variation of the two signals did not exceed 2 mV. The demodulated angle is shown in Figure 10c, and the fluctuation did not exceed 0.002°. The main cause of the fluctuation in the long-term stability test was the environmental impact, especially the vibration acting on the sensitive structure.

#### 4.2.2. Step Test

**Figure 11:**
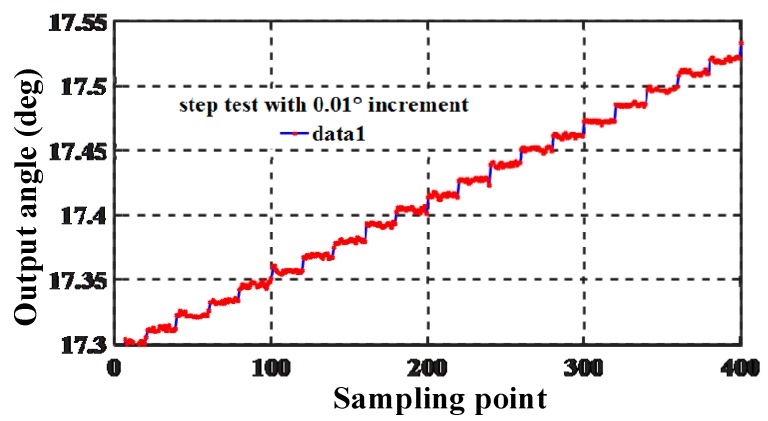
Output response of the step test with 0.01° increments.

In order to verify the sensitivity of the ASIC and sensitive structure, the step size was tested. To determine the resolution of the proposed encoder, a step test with an increment of 0.01° and delay of 2.5 s was performed. The corresponding output angle response is shown in Figure 11. The resolution of the proposed angle sensor was considerably better than 0.01°; thus, it meets most application requirements.

#### 4.2.3. Linearity Test

**Figure 12:**
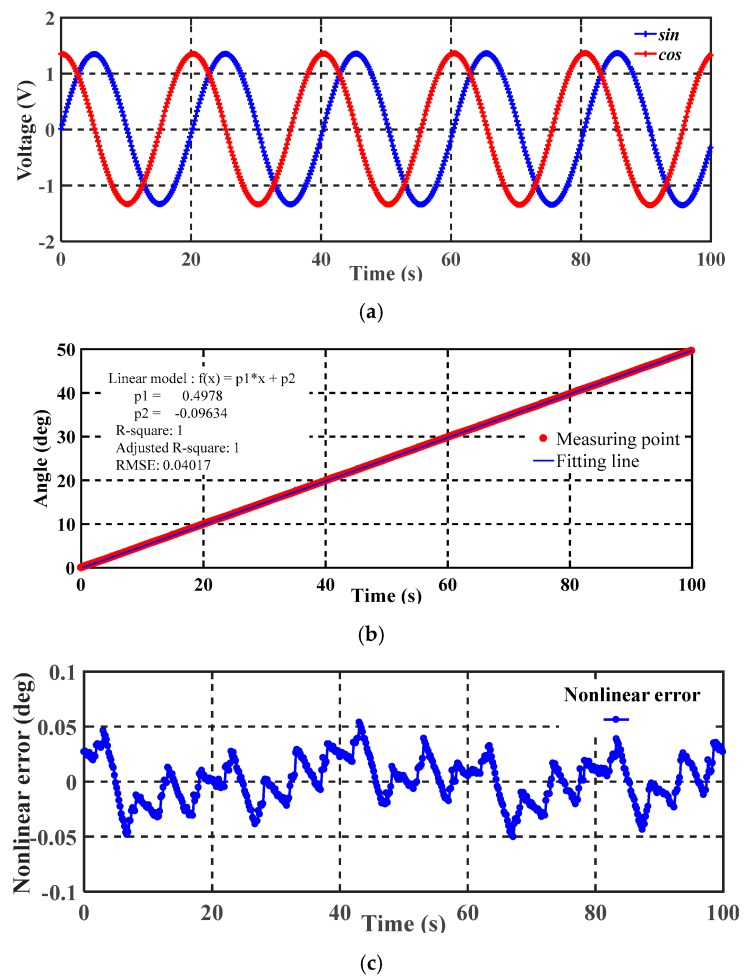
Linearity test results: (**a**) two amplitude modulated signal outputs without the DC and carrier signal, (**b**) demodulated angle value, and (**c**) the corresponding nonlinear error of (**b**).

In this test, two orthogonal amplitude modulated signals $U_{S}$ and $U_{C}$ were collected and drawn, as shown in Figure 12a. The output voltage approximately varied from 1 V to −1 V, and the two signals were orthogonal to the theoretical output. The measurement data verified the correctness of the sensor processing of the sensitive structure and the backend ASIC design. The angular accuracy was further compared. The two signals were inversely cut to obtain the angle information shown in Figure 12b, and the linear characteristics were compared. The measured integral nonlinearity error is ±0.05°. The main reasons which caused the nonlinear error are installation errors and manufacturing errors.

### 4.3. Summary of the Encoder

The performance of the analog-interfacing circuit and angle encoder are summarized in Table 1. A capacitance resolution of 10^−19^ F was achieved under DC conditions. The test results showed that the angle stability was up to 0.003°, the accuracy was 0.05°, and the power consumption was less than 20 mW. The main reason for the limited accuracy was that the frontend ASIC circuit had a large 1/*f* noise. The proposed device has the advantages of small volume and low power consumption and has the outstanding potential for use in portable electronics, robot arms and aerial heads. For more flexible expansion, the proposed circuit can be simply combined with a low-powered microcontroller unit (MCU) with dual ADC (e.g., Atmel ATSAML22G), for accurate angle calculation. The proposed circuit has a wide application range and high market demand.

## 5. Conclusions

This paper proposes an analog interface circuit for a capacitive angle encoder. Based on the capacitance elimination array and switch synchronous demodulation method, the circuit combines with a sensitive structure to achieve a measurement resolution of better than 0.01° and precision of better than ±0.05°. Test and measurement results confirmed that the integrated system worked properly. The proposed analog-interfacing circuit effectively solves the problem of high power consumption and large volume of the encoder demodulation circuit. Encoders with the proposed analog interface circuit can be widely used in UAVs, small robots, high-precision gimbal systems, etc., because of its low power consumption and easy integration.

## Figures and Tables

**Table 1:** Performance of the analog-interfacing circuit and angular encoder.

Properties	Values
Process technology	Smic 0.18 µm CMOS
Supply voltage	5 V
Scale factor	4 V/pF
Excitation frequency	250 kHz
Eliminated capacitance	Max.: 12495 fF Min.: 49 fF
Max. nonlinearity	0.06% FS
Noise floor	400 nV $\sqrt{Hz}$
Power consumption	<20 mW
Chip area	3 mm^2^
Resolution	<0.01°
Stability	0.002°
Precision	±0.05°
